# Multi-omics unveils tryptophan metabolic pathway as a key pathway influencing residual feed intake in Duroc swine

**DOI:** 10.3389/fvets.2024.1403493

**Published:** 2024-05-29

**Authors:** Shujie Wang, Dong Chen, Xiang Ji, Qi Shen, Yang Yu, Pingxian Wu, Guoqing Tang

**Affiliations:** ^1^Farm Animal Genetic Resources Exploration and Innovation Key Laboratory of Sichuan Province, Sichuan Agricultural University, Chengdu, China; ^2^State Key Laboratory of Swine and Poultry Breeding Industry, Sichuan Agricultural University, Chengdu, China; ^3^National Center of Technology Innovation for Pigs, Rongchang, Chongqing, China; ^4^Chongqing Academy of Animal Sciences, Rongchang, Chongqing, China

**Keywords:** pigs, feed efficiency, residual feed intake, metabolomics, gut microbiota

## Abstract

The genetic trait of residual feed intake (RFI) holds considerable importance in the swine industry. Recent research indicates that the gut microbiota of pigs plays a pivotal role in the manifestation of the RFI trait. Nevertheless, the metabolic pathways involved in the functioning of these microorganisms remain elusive. Thus, based on the ranking of the RFI trait in Duroc pigs, the present study selected the top 10 and bottom 10 pigs as the experimental subjects. The distribution and metabolite differences of cecal microbiota were analyzed using 16S rRNA gene sequencing and liquid chromatography–tandem mass spectrometry (LC–MS/MS) techniques. The low RFI cecal group was named LRC, and the high RFI cecal group was named HRC. The results indicate that the LRC group had lower RFI, feed conversion ratio (FCR), average daily feed intake (ADFI) (*p* < 0.001), and thinner backfat (*p* < 0.05) compared with the HRC group. We simultaneously recorded the foraging behavior as well, the LRC group had a significant increase in total time spent at the feeder per day (TPD) (*p* < 0.05) and a significant increase in average feed intake per mins (AFI) and the number of visits to the feeder per day (NVD) compared to the HRC group (*p* < 0.001). *Clostridium_XVIII*, *Bulleidia*, and *Intestinimonas* were significantly enriched in the LRC group (*p* < 0.01), while *Sutterella*, *Fusobacterium*, and *Bacteroides* were significantly increased in the HRC group (*p* < 0.01). In the metabolome, we detected 390 (248 metabolites up and 142 down in the LRC compared with HRC), and 200 (97 metabolites up and 103 down in the LRC compared with HRC) differential metabolites in positive and negative ionization modes. The comprehensive analysis found that in the LRC group, *Escherichia* and *Eubacterium* in the gut may increase serotonin content, respectively. *Bacteroides* may deplete serotonin. We suggest that the RFI may be partly achieved through tryptophan metabolism in gut microbes. In individuals with low RFI, gut microbes may enhance feed efficiency by enhancing host synthesis and metabolism of tryptophan-related metabolites.

## 1 Introduction

In animal production, the indicators we evaluate for animal feed efficiency (FE) are usually feed conversion ratio (FCR) and residual feed intake (RFI). FCR measures how much feed is required for each pound of weight gain, while RFI measures the feed intake of an animal compared to its expected feed intake based on its weight and rate of gain (1). However, FCR is a ratio-based metric, which means that pigs with different growth and development status can have the same value, leading to errors in breeding outcomes (2). This limits the effectiveness of using FCR as a sole measure of feed efficiency in swine production. A more comprehensive approach would be to use additional measures such as RFI, which evaluates an animal’s feed intake relative to their expected feed intake based on individual factors such as weight, backfat, and growth rate (3). By utilizing multiple metrics, a better understanding of feed efficiency in swine production can be achieved, resulting in more accurate and effective strategies.

The gut microbiome is a complex system that plays a crucial role in the overall health and well-being of mammals. This intricate ecosystem is made up of trillions of microorganisms that live within the gastrointestinal tract and form a symbiotic relationship with the host animal. The gut microbiome serves a myriad of functions within the body, including metabolism (4, 5) and immune system regulation (6), nutrient absorption (7), and protection from pathogens (8). In terms of feed efficiency, gut microbiota plays a crucial role by degrading complex compounds in the food, synthesizing vitamins (9), regulating amino acid metabolism (10), and facilitating mineral absorption (11), among other pathways, to provide the host with essential nutrients. Noteworthy findings have recently emerged indicating that the cecal microbiota of low RFI pigs displays increased prevalence of *Escherichia/Shigella*, *Ruminobacter*, and *Veillonella*, whereas high RFI pigs exhibit heightened colonization of *Campylobacter* (12). Among Duroc pigs, various members of *Clostridia* were found to be notably enriched in individuals with low RFI, while bacteria linked to inflammation, such as *Prevotella*, exhibited higher abundance in individuals with high RFI (13). These discoveries imply a correlation between gut microbiota and feed efficiency in pigs, although the exact mechanisms remain elusive.

The microbial population relies on the nutrient supply of the host to maintain its growth and population stability, while the host benefits from the metabolic activities of the microbial population and regulates its own functions by digesting and utilizing its metabolites. Previous investigations have elucidated that the cecal microbiota synthesizes diverse metabolites, which are subsequently absorbed by the cecal mucosal epithelial cells, thereby influencing the efficiency of nutritional absorption and resulting in phenotypic variations (14–16). After transplanting microbiota from low RFI chickens into high RFI chickens, a noticeable disparity in the profile of short-chain fatty acids in the cecal microbiome composition was observed (17). Nevertheless, there are limited studies on the complex relationship between the cecal microbial porcine RFI phenotype and the cecal microbiota and metabolites.

In addition, feeding behavior, one of the important cornerstones of life activities, has been found to affect representations with feed efficiency (18, 19). Yang et al. found that selection for decreased RFI has resulted in pigs that spend less time eating and eat faster (20). Research indicates that there is a positive genetic and phenotypic correlation (0.95 and 0.90, respectively) between daily average feed intake and RFI (21). Hence, exploring the correlation between feeding behavior and RFI can aid us in gaining profound insights into the food intake process and further optimizing feeding management measures to enhance animal growth efficiency and overall health status.

In the current commercial pig breeding systems, Duroc pigs are commonly selected as superior sires in the Duroc-Landrace-Yorkshire crossbreeding program. Compared to Landrace and Yorkshire pigs, Duroc pigs are known for their outstanding on feed efficiency and growth rate. Therefore, studying the functionality of their gut microbiota in relation to feed utilization efficiency holds significant importance. The objective of this study is to establish correlations between RFI phenotypes and cecal microbiota and metabolites in Duroc pigs, with a particular focus on differences in feeding behavior among individuals with extreme RFI. The aim is to provide insights that can contribute to enhancing feed efficiency.

## 2 Materials and methods

### 2.1 Animals

In this study, 209 Duroc gilts were generously donated by a commercial pig farm located in Sichuan, China, with an average age of 83.62 ± 5.37 days and an average weight of 33.68 ± 4.23 kg. The Duroc gilts were randomly allocated to 15 pens, with 12–14 pigs per pen, and each pen was equipped with a state-of-the-art Feed Intake Recording Equipment (FIRE) (RLX-096, Osborne, United States). Prior to the onset of the experiment, the pigs were allowed to acclimatize to the FIRE equipment over a duration of 1 week. The measurement process lasted for a total of 93 days, and each pig was assigned a unique radio frequency identification tag on the left ear for the FIRE system to detect. Upon entering the FIRE, the system automatically recorded pertinent parameters, including the number of visits to the feeder, feeding time, feed intake, and body weight. The swine resided within a climate-controlled environment, the temperature modulating between a mild 18 and a warm 25 degrees Celsius, accompanied by a relative humidity oscillating from 60% to a slightly damp 80%, with a 12 h light/dark cycle (lighting from 7 am to 7 pm). Throughout the trial, all the pigs were fed with the same corn-soybean commercial diet, conforming to the Chinese standard GB/T 5915-2020, and were not administered antibiotics or drugs. The primary constituents of the feed comprise maize, soya bean meal, and bran. It boasts a composition of 15% crude protein, 1.6% crude fat, 5% crude fiber, and 7.5% crude ash, in addition to noteworthy amounts of lysine (0.9%), calcium (0.8%), phosphorus (0.6%), and a dash of salt at 0.3%. Adequate supplies of clean water were provided *ad libitum* during the entire study period, and regular veterinary inspections were conducted to ensure that only healthy animals were selected for the trial.

### 2.2 Data collection and RFI calculation

We performed data quality control on the information gathered by the FIRE system, which were evaluated based on certain parameters: daily feed intake ranging from 0.5 to 4.5 kg, the number of feed intakes per day of 2 to 20 times, and daily feeding time between 5 min to 2 h. Any data that fell outside of these established ranges were identified as outliers and subsequently removed. After quality control, the dataset had reduced from the initial 105,202 entries to 92,305 entries. Each day’s weight was calculated based on the average measured body weight. Using the data that passed the quality control test, we then evaluated each pig’s total time spent at the feeder per day (TPD), number of visits to the feeder per day (NVD), average feed intake per minutes (AFI), measurement initiation body weight (W1), measurement termination body weight (W2), and average daily feed intake (ADFI). At the point when the pigs’ average weight reached 115 kg, their backfat thickness (BFT) in the tenth rib was determined using real-time B-mode ultrasound (MyLab^™^X7, ESAOTE, Genova, Italy).

The average daily gain (ADG), ADFI, FCR, and RFI were calculated by the following model (22, 23):


$$ADG=\frac{W2-W1}{\text{test}\;\text{days}}$$

$$\text{ADFI}=\frac{TFI}{\text{test}\;\text{days}}$$

$$FCR=\frac{TFI}{W2-W1}$$

$$AMW=\frac{\left(W2^{1.6}-W1^{1.6}\right)}{1.6\times \left(W2-W1\right)}$$

RFI=ADFI−14.1ADG−2.83BFT−110.9AMW

Where TFI refers to total feed intake. AMW means average metabolic weight.

### 2.3 Sample collection

The HRC and LRC groups were comprised of the ten pigs with the highest and lowest RFI scores, respectively. Twenty select pigs were then transported to a commercial slaughterhouse for slaughter, where they remained on-site overnight, and fasted but with *ad libitum* access to water prior to being slaughtered the following morning. The selected live pigs were humanely slaughtered in accordance with the Live Pig Slaughter Guidelines (GB/T 17236-2019), which are approved by the General Administration of Quality Supervision, Inspection, and Quarantine of the People’s Republic of China and the Standardization Administration of the People’s Republic of China. After being stunned with carbon dioxide, they were bled for slaughter. The cecum was opened with sterile ophthalmic scissors, and the cecal contents were carefully collected with sterile forceps, immediately transferred to a sterile 50 mL centrifuge tube, and preserved in liquid nitrogen. Separate utensils were employed for each sample to ensure that there was no cross-contamination.

### 2.4 16S rRNA gene sequencing

The QiAamp DNA Stool Mini Kit (Qiagen, Hilden, Germany) was employed to extract DNA from the cecal contents in line with the manufacturer’s instructions. Subsequently, approximately 30 ng of genomic DNA samples were taken and matching fusion primers were employed to establish an amplification PCR reaction system. The PCR amplification products were then purified with Agencourt AMPure XP magnetic beads (Beckman Coulter, Brea, CA), dissolved in Elution Buffer, labelled, and subjected to library construction. The fragment range and concentration of the library were determined using the Agilent 2,100 Bioanalyzer (Agilent, Santa Clara, CA, United States). Using the HiSeq platform, properly built libraries were sequenced based on the insert size. For sequence assembly, we used the Fast Length Adjustment of Short reads (FLASH) splicing program (v.1.2.11) to join the paired reads generated from paired-end sequencing into one sequence using overlapping relationships to produce tags of hypervariable regions (24). The splicing conditions were set such that the minimum match length was 15 bp, and the allowable mismatch rate in the overlapping region was 0. Afterwards, the spliced Tags were clustered into Operational Taxonomic Units (OTU s) (sequence similarity >97%) utilizing USEARCH (v.7.0.1090) software (25).

### 2.5 16S rRNA gene sequencing data analysis

Calculation of the Alpha diversity analysis (including Chao1 index, Ace index, Shannon index, Simpson index and the Good’s coverage) was performed using MOTHUR (v.1.30.1) (26). Beta-diversity was quantified through the utilization of the Bray–Curtis distance metric, which was computed as resemblances (ANOSIM). Furthermore, the visualization of the results was accomplished by employing the QIIME2 package in the R software, employing principal coordinates analysis (PCoA) (27). Linear discriminant analysis effect size (LEfSe) analysis was performed using LEfSe software (28). PICRUSt2 (v.2.2.0b) was used for functional annotation of microbial communities (29). The rest of the graphs were drawn using the R (v 4.1.0). Statistically significant variations in the abundance of microbiota were observed at the phylum and genus levels, distinguishing between the HRC and LRC groups. This discrimination was determined by employing Student’s *t*-test and controlling for false discovery rate (FDR). Based on the 16S sequencing data, functional predictions based on the Kyoto Encyclopedia of Genes and Genomes (KEGG) database were performed using the PICRUSt2 software (30).

### 2.6 Liquid chromatography tandem mass spectrometry (LC–MS/MS) analysis

In this experiment, approximately 60 mg of cecal contents were extracted from each of the cryopreserved HRC and LRC groups. Ground the samples into homogenate using a tissue grinder. Then, added 400 mL of extraction solvent (methanol: water = 4:1) for extraction. Collected the supernatant by centrifugation (13,000 g, 15 min, 4°C) for further analysis. To separate and detect metabolites, a Waters 2D UPLC system (provided by Waters Corporation, United States) in combination with a tandem Q Exactive high-resolution mass spectrometer (manufactured by Thermo Fisher Scientific, MA, United States) was employed. The following chromatographic conditions were applied: employment of a BEH C18 column (100 mm length, 2.1 mm internal diameter, and 1.7 μm particle size; Waters, Milford, United States); the mobile phase for positive ion mode entailed an aqueous solution containing 0.1% formic acid (Solution A) and 100% methanol comprising 0.1% formic acid (Solution B). Conversely, the mobile phase for negative ion mode utilized an aqueous solution harboring 10 mM ammonium formate (Solution A) and 95% methanol containing 10 mM ammonium formate (Solution B). The elution was executed along the following gradient: 0–1 min, 2% Solution B; 1–9 min, 2–98% Solution B; 9–12 min, 98% Solution B; 12–12.1 min, a transition from 98% Solution B to 2% Solution B; 12.1–15 min, 2% Solution B. The sample injection volume was set at 5 μL, and the flow rate reached 0.36 mL/min. The column temperature was maintained at 45°C. Moreover, both positive and negative ion scan modes were employed to capture the signal emanating from the mass spectrometer analysis. Regarding the mass spectrometry settings, a stepped normalized collision energy protocol was utilized, employing values of 20, 40, and 60 eV respectively, while the spray voltage was set to 3.80 KV for positive mode and 3.20 KV for negative mode. Additional settings encompassed a capillary temperature of 320°C, an auxiliary gas heater temperature of 350°C, a sheath gas flow rate of 40 mL/min, and an auxiliary gas flow rate of 10 mL/min.

### 2.7 LC–MS/MS data analysis

The raw data were preprocessed by metaX software for data preprocessing, statistical analysis, metabolite classification annotation, and functional annotation (31). The analysis was conducted utilizing the free online platform MetaboAnalyst 5.0^1^ (32). Calculate the Variable importance in the projection (VIP) value from Partial Least Squares-Based Discriminant Analysis (PLS-DA). VIP ≥ 1.0, absolute fold change (FC) ≥ 2.0, *p* < 0.05 (Student’s *t*-test) were used as criteria for the selection of differential metabolites. The rest of the graphs were drawn using the R software (v.4.1.0). Pearson correlation analysis was used to determine the relationship between microbial communities and metabolites using the heatmap package (v.1.0.12) in R software.

### 2.8 Combined analysis of microbiome and metabolome

The prediction of the heat map relied on a genome-scale metabolic model (GEM)-derived prognostic model. This prognostic model was built upon a logical regression model, trained on a meticulously crafted, GEM. It holed the capacity to anticipate the potential synthesis of each metabolite across various categorical tiers.

### 2.9 Statistical analysis

The data regarding growth performance and feeding behavior measurements were expressed as the mean ± standard deviation (SD). Statistical comparisons between groups were conducted using Student’s *t*-test with the aid of SPSS (v.22.0). For all results, the *P* criterion was as follows: *p < 0.05 was determined to be statistically significant, **p < 0.01 indicated a high level of statistical significance, and ***p < 0.001 denoted extremely significant.

## 3 Results

### 3.1 Growth performance and feeding behavior of pigs

First, we conducted statistical analysis on the production performance of the selected pigs (Table 1). The significant differences in RFI phenotypes prove the success of our grouping (*p* < 0.05). The results revealed that the LRC group exhibited lower FCR, ADFI (*p* < 0.001), and lower thicker backfat (*p* < 0.05) compared to HRC group.

**Table 1:** Ranking pigs by RFI statistics on growth performance parameters and feeding behavior.

Parameter	LRC (*n* = 10)	HRC (*n* = 10)	*p*-value
RFI (kg)	−0.270 ± 0.020	0.353 ± 0.017	< 0.001
FCR	1.971 ± 0.104	2.422 ± 0.191	< 0.001
ADFI (kg/day)	1.863 ± 0.125	2.358 ± 0.190	< 0.001
ADG (kg/day)	0.946 ± 0.067	0.975 ± 0.052	0.328
BFT (mm)	8.365 ± 1.559	9.857 ± 1.225	0.037
TPD	65.413 ± 3.613	59.983 ± 4.549	0.015
NVD	5.685 ± 0.643	7.265 ± 0.486	< 0.001
AFI	0.029 ± 0.003	0.039 ± 0.004	< 0.001

Mean and standard deviation (SD) concentrations were given as the mean ± SD. RFI, residual feed intake; FCR, feed conversion ratio; ADFI, average daily feed intake; ADG, average daily gain; BFT, backfat thickness; TPD, total time spent at feeder per day; NVD, number of visits to the feeder per day; AFI, average feed intake per mins. The *P* were determined using student’s *t*-test.

Additionally, we assessed the feeding behavior of both groups, primarily focusing on the following metrics: TPD, NVD, and AFI. Interestingly, the LRC group exhibited markedly elevated levels of TPD (*p* < 0.05), contrasted by considerably and highly significantly reduced measures of AFI and NVD (*p* < 0.001) when juxtaposed with the HRC group.

### 3.2 Sequencing analysis and alpha and beta diversity

A total of 1,111,597 readings were obtained from twenty specimens, averaging 55,579 readings per specimen. Following a thorough quality control and screening process, 1,288 OTUs were identified.

We have demonstrated the disparity in alpha diversity between the two groups, examining two facets: calculating the indices of bacterial richness estimators (Chao1, Ace) and computing the indices of bacterial diversity (Shannon, Simpson). The corresponding outcomes have been elaborated in Table 2. Notably, when comparing the LRC group to the HRC group, the flora’s abundance exhibited a significant difference (*p* < 0.01), whereas disparities in flora diversity were not statistically significant. Furthermore, the Good’s coverage analysis revealed a comprehensive coverage exceeding 99%, thus affirming the reliability of the findings.

**Table 2:** Differences in the cecal contents microbial diversity of the two groups.

Parameter	LRC (*n* = 10)	HRC (*n* = 10)	*p*-value
Chao1	723.45 ± 122.62	574.44 ± 73.00	< 0.01
Ace	713.56 ± 108.14	554.66 ± 68.16	< 0.01
Shannon	3.96 ± 0.63	3.40 ± 0.45	0.063
Simpson	0.07 ± 0.06	0.11 ± 0.06	0.315
Coverage	0.99 ± 0.00	0.99 ± 0.00	0.063

The data were expressed as the mean values ± standard deviation (SD). The *P* were determined using student’s *t*-test.

**Figure 1:**
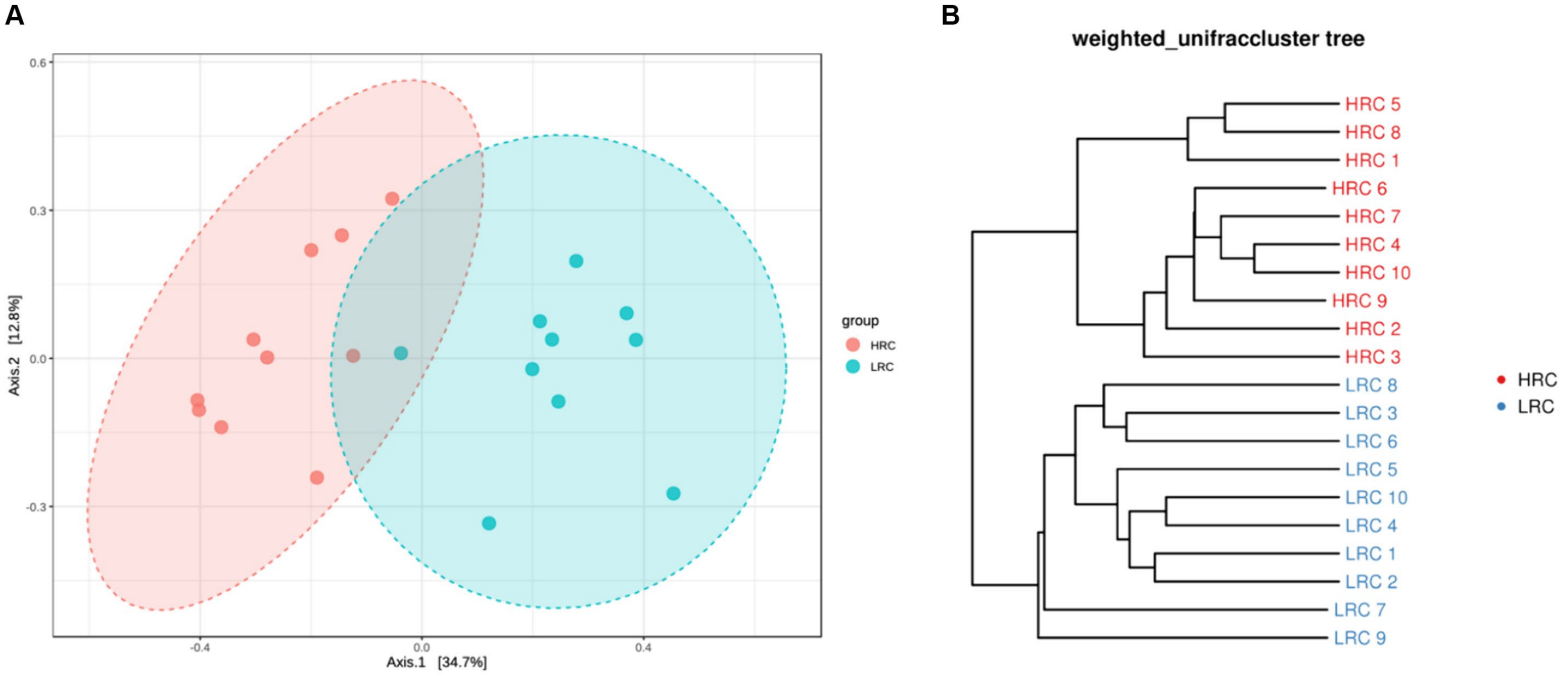
Differences in beta diversity between the two groups. **(A)** Principal Co-ordinates Analysis (PCoA) at the OTUs level. Varied colors represent different classifications, and a closer proximity amongst the samples indicates a higher similarity in their microbial compositions, thereby inferring a diminished degree of variance. **(B)** Based on the distance matrix of beta diversity, hierarchical clustering analysis, utilizing the NPGMA (Unweighted Pair Group Method with Arithmetic Mean) algorithm, was performed to construct a dendrogram, yielding a tree-like relationship for visual analytical purposes.

In the beta diversity difference results, PCoA revealed distinct structural differences in the composition of the intestinal microbiota between the LRC and HRC group (*p* < 0.05) (Figure 1A). In light of the OTU analysis results derived from each sample, the widely utilized weighted unifrac index will be implemented to measure the dissimilarity coefficient between two specimens. As depicted in Figure 1B, a pronounced separation had been observed amongst the samples from both groups.

### 3.3 Microbial composition

**Figure 2:**
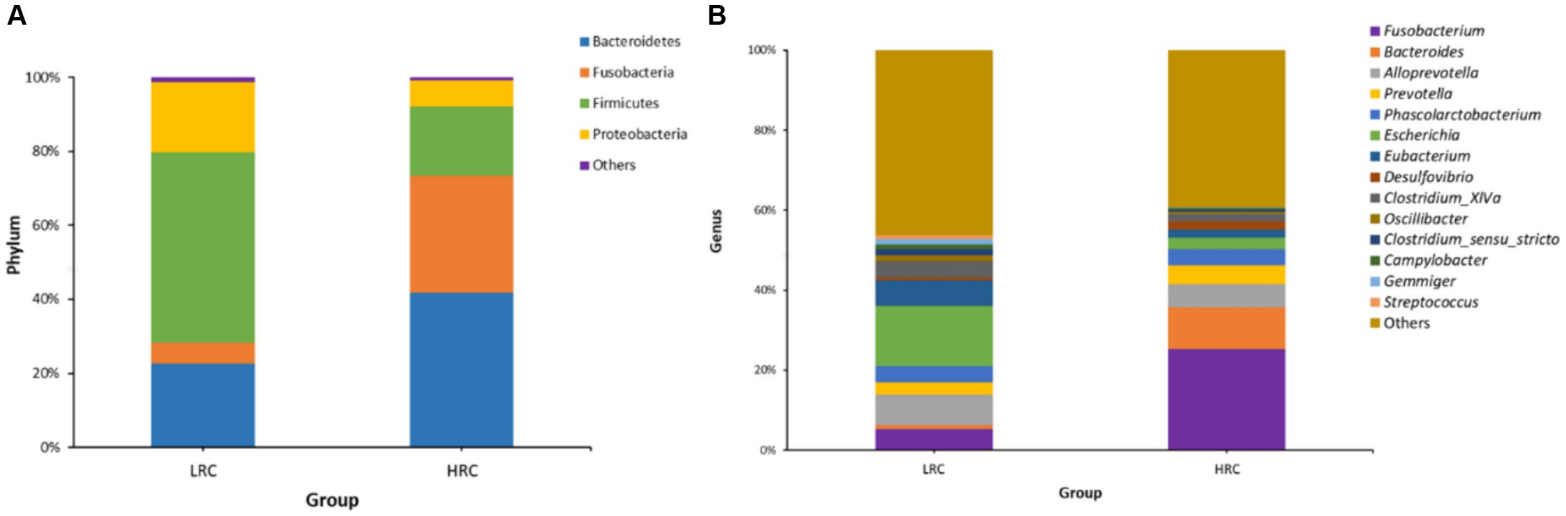
Microbial composition of gut microbiota in experimental pigs at different taxonomic levels by 16S rRNA gene sequencing. **(A)** The overall compositions of the cecal contents microbiota of the LRC and HRC groups were represented as bar plots at the phylum level. **(B)** The overall compositions of the cecal contents microbiota of the LRC and HRC groups were represented as bar plots at the genus level.

In the LRC group, three phyla, namely Bacteroidetes (22.53%), Firmicutes (51.28%), and Proteobacteria (19.19%), were dominant. Conversely, in the HRC group, Bacteroidetes (41.48%), Fusobacteria (32.45%), and Firmicutes (18.28%) played a pivotal role (Figure 2A). On a genus level analysis, the LRC group presented four prevailing genera: *Escherichia* (15.18%), *Alloprevotella* (7.69%), *Eubacterium* (6.78%), and *Fusobacterium* (5.26%). In comparison, the four dominant genera within the HRC group comprised of *Fusobacterium* (25.80%), *Bacteroides* (10.52%), *Alloprevotella* (5.51%), and *Prevotella* (4.64%) (Figure 2B).

As delineated in Table 3, we discerned six, nine, nine, and six markedly disparate microorganisms at the ranks of phylum, class, order, family, and genus correspondingly, each substantiating a FDR less than 0.05 (all differential microorganisms were shown in Supplementary Table S1).

**Table 3:** Significant microbial differences in the cecal contents microbial of the two groups.

Level	Name	*p*-value	FDR	Statistics (LRC VS HRC)
Phylum	Firmicutes	0.000	0.000	6.255
	Proteobacteria	0.017	0.041	2.813
	Actinobacteria	0.019	0.041	2.809
	Lentisphaerae	0.006	0.021	−3.479
	Bacteroidetes	0.002	0.008	−3.652
	Fusobacteria	0.001	0.008	−4.246
Class	Clostridia	0.000	0.002	5.954
	Erysipelotrichia	0.004	0.019	3.762
	Gammaproteobacteria	0.013	0.032	3.008
	Actinobacteria	0.019	0.042	2.809
	Deltaproteobacteria	0.008	0.022	−3.153
	Betaproteobacteria	0.005	0.021	−3.349
	Oligosphaeria	0.006	0.022	−3.479
	Bacteroidia	0.002	0.011	−3.700
	Fusobacteriia	0.001	0.011	−4.246
Order	Clostridiales	0.000	0.002	5.955
	Erysipelotrichales	0.004	0.021	3.762
	Enterobacteriales	0.013	0.036	3.000
	Coriobacteriales	0.019	0.046	2.809
	Desulfovibrionales	0.007	0.022	−3.251
	Burkholderiales	0.005	0.022	−3.349
	Oligosphaerales	0.006	0.022	−3.479
	Bacteroidales	0.002	0.012	−3.700
	Fusobacteriales	0.001	0.012	−4.246
Family	Ruminococcaceae	0.002	0.025	4.168
	Erysipelotrichaceae	0.004	0.028	3.762
	Lachnospiraceae	0.005	0.028	3.506
	Eubacteriaceae	0.007	0.028	3.284
	Enterobacteriaceae	0.013	0.045	3.000
	Rikenellaceae	0.014	0.045	−2.835
	Desulfovibrionaceae	0.007	0.028	−3.251
	Sutterellaceae	0.005	0.028	−3.349
	Oligosphaeraceae	0.006	0.028	−3.479
	Fusobacteriaceae	0.001	0.021	−4.246
	Bacteroidaceae	0.000	0.006	−5.584
Genus	*Clostridium_XVIII*	0.003	0.047	3.783
	*Bulleidia*	0.002	0.047	3.748
	*Intestinimonas*	0.002	0.047	3.689
	*Sutterella*	0.004	0.047	−3.434
	*Fusobacterium*	0.002	0.047	−3.872
	*Bacteroides*	0.000	0.014	−5.584

The *P* were determined using student’s *t*-test. FDR values were determined by Benjamini–Hochberg FDR correction. The sample size was *n* = 10.

**Figure 3:**
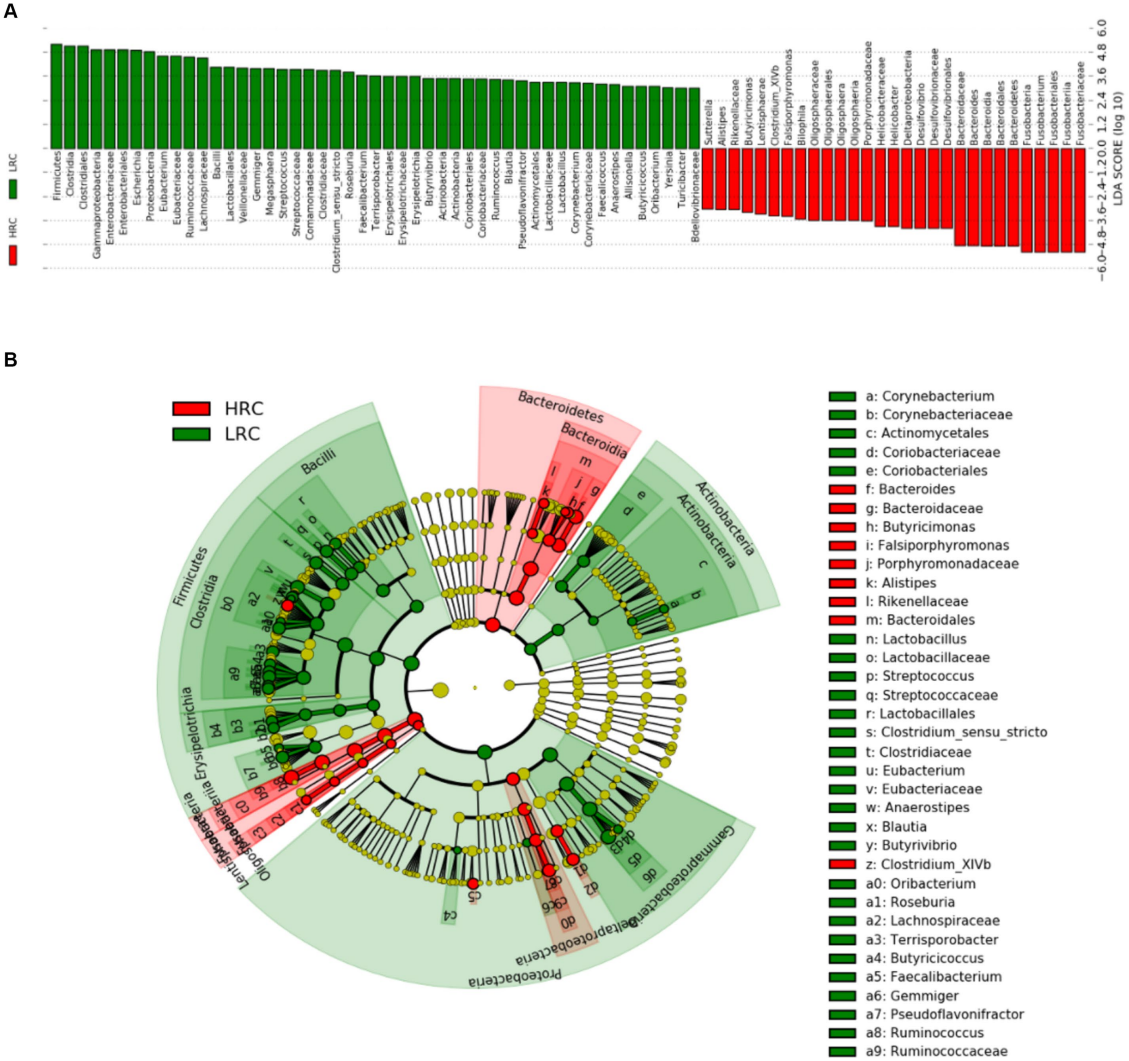
LEfSe classification. LEfSe analysis revealed a histogram **(A)** and a dendrogram **(B)**, depicting the gut bacteria of utmost biological significance in each group. In both the Kruskal–Wallis and paired Wilcoxon tests, *p* < 0.05 and LDA ≥ 3.0 were considered significant at a significance level of 0.05. The sample size was *n* = 10.

Linear discriminant analysis scoring investigation has facilitated the detection of statistically substantial biomarkers amidst the groups, namely species exhibiting conspicuous dissimilarities across said groups. In the non-parametric Kruskal–Wallis rank sum test, the LEfSe analysis conducted at a threshold level of LDA ≥ 3.0 and *p* < 0.05 revealed 78 pathways that explained the distinguishing features between HRC and LRC pigs (Figure 3).

### 3.4 Microbial functional enrichment analysis

To explore into the functionalities of these distinctive microorganisms, we conducted KEGG pathway analyses using PICRUSt2. Results of considerable divergence were identified when mean relative abundances exceeded 1%, accompanied by *P* and FDR values below 0.05. A total of 18 KEGG pathways exhibited noteworthy dissimilarities between the LRC and HRC groups, as illustrated in Table 4.

**Table 4:** KEGG functional enrichment analysis in the microbiome.

Function	Log_2_FC	*p*-value
Biosynthesis of ansamycins	0.42	0.00
D-Glutamine and D-glutamate metabolism	0.20	0.01
Fatty acid biosynthesis	0.17	0.05
Phenylalanine, tyrosine and tryptophan biosynthesis	0.17	0.02
Tryptophan metabolism	0.16	0.00
Histidine metabolism	0.16	0.05
Selenocompound metabolism	0.08	0.00
Peptidoglycan biosynthesis	0.08	0.00
Pantothenate and CoA biosynthesis	0.08	0.01
Cysteine and methionine metabolism	0.08	0.00
Folate biosynthesis	0.07	0.00
Streptomycin biosynthesis	−0.09	0.00
Protein export	−0.16	0.03
Lysine biosynthesis	−0.18	0.02
Biosynthesis of vancomycin group antibiotics	−0.22	0.00
Valine, leucine and isoleucine biosynthesis	−0.40	0.01
Biotin metabolism	−0.41	0.00
Pentose phosphate pathway	−0.53	0.00

The *P* were determined using Wilcoxon Rank-Sum Test. FC, fold changes (LRC VS HRC). Log_2_FC means taking the base 2 logarithm of FC.

Importantly, the LRC group epitomized elevated activity throughout pathways encompassing annamycin, pantothenate, CoA, and folate synthesis, as well as the formation of fatty acids, phenylalanine, tyrosine, and tryptophan. Furthermore, a marked amplification was noted in the metabolism of D-glutamine and D-glutamate, tryptophan, histidine, selenocompound, cysteine, and methionine.

Conversely, within the HRC group, there was a conspicuous augmentation in the relative abundance of streptomycin, lysine, valine, leucine, and isoleucine biosynthesis, protein transport mechanisms, the genesis of vancomycin group antibiotics, biotin metabolism, and the pentose phosphate pathway.

### 3.5 Differential metabolite analysis

**Figure 4:**
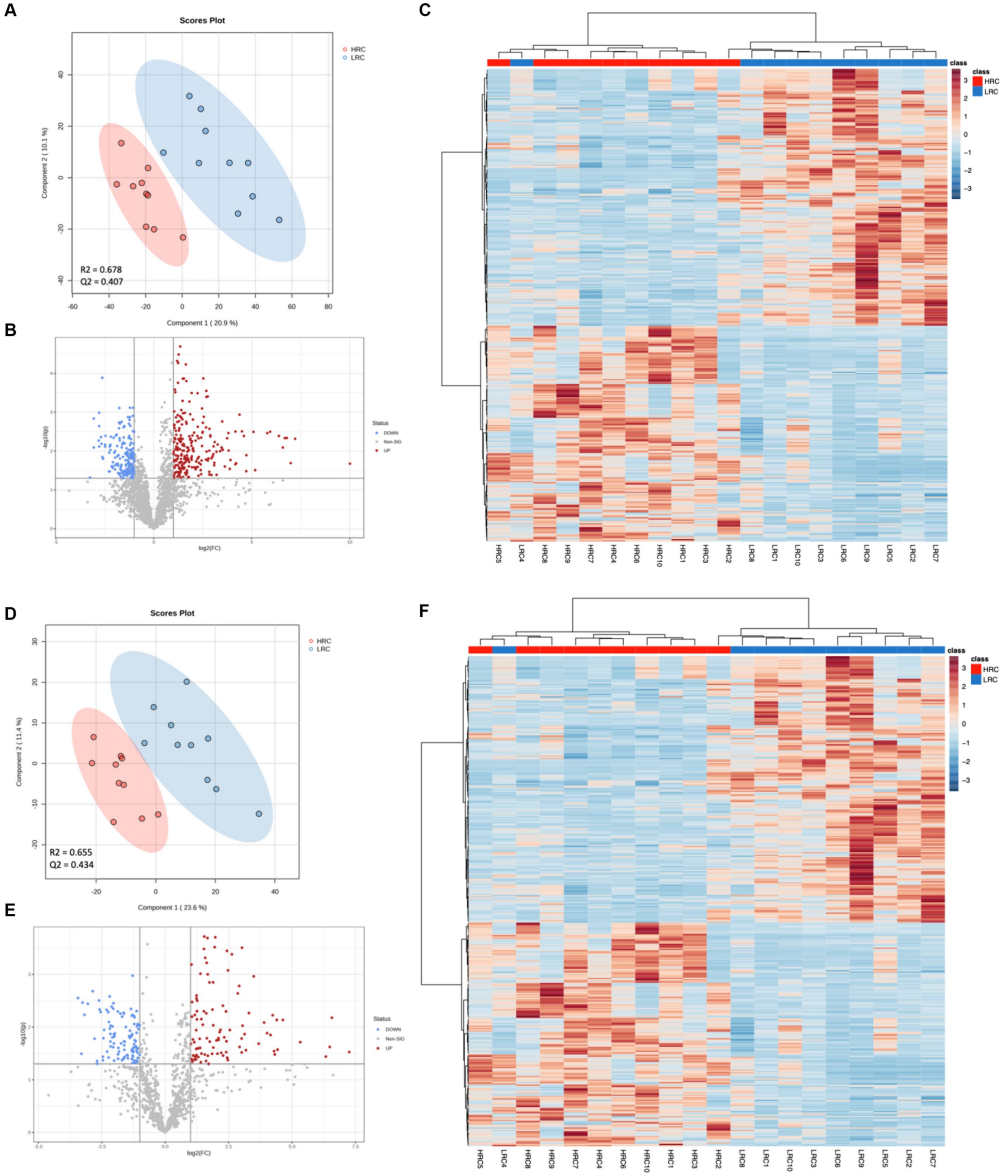
Significant differences in metabolites between LRC and HRC groups. **(A,D)** Scatter plot illustrating the scores of Partial Least Squares-Based Discriminant Analysis (PLS-DA). **(B,E)** Volcano plots portraying the differential accumulation of metabolites. **(C,F)** Heatmap featuring the differential expression of metabolites, where the color red represented high abundance in the HRC group, while blue represented high abundance in the LRC group. **(A–C)** in positive ionization mode and **(D–F)** in negative ionization mode.

We performed a metabolomic analysis of cecal contents to understand how metabolites differed between the two groups. The 6,838 and 1,667 m/z features were detected in positive and negative ionization modes, respectively. We used the PLS-DA model to identify differential metabolites between the two groups. The parameters of the models: *R*^2^ = 0.664 *Q*^2^ = 0.382 for positive ionization mode; *R*^2^ = 0.671 *Q*^2^ = 0.426 for negative ionization mode (Figures 4A,D). The results showed that there was a clear separation between the two groups. Based on the criteria of *p* < 0.05, |Log_2_FC| > 1, VIP > 1, we detected 390 (248 metabolites up and 142 down), 200 (97 metabolites up and 103 down) differential metabolites in positive and negative ionization modes (Figures 4B,E).

The top ten metabolites, which expressed prominent divergence in their levels, were elucidated in Table 5. Detailed metabolite information was listed in Supplementary Table S2. In addition, we did cluster analysis on the differential metabolite, and the findings showed good cluster quality (Figures 4C,F).

**Table 5:** Top 10 leading metabolites that have been identified with noteworthy disparities.

Name	ESI	*p*-value	Log_2_(FC)	VIP
4-(2-aminophenyl)-2,4-dioxobutanoic acid	NEG	0.030	7.254	1.323
Xanthurenic acid	POS	0.005	7.060	1.763
4-hydroxy-2-quinolinecarboxylic acid	POS	0.024	6.848	1.460
2-(carboxyacetamido)benzoic acid	POS	0.005	6.657	1.766
3-(2-oxo-2,3-dihydro-1,3-benzoxazol-3-yl) propanoic acid	POS	0.006	6.441	1.715
N-acetylsphingosine	NEG	0.005	−2.308	1.646
5beta-scymnol	POS	0.000	−2.392	2.170
Proscillaridin	NEG	0.011	−2.408	1.507
Cedefingol	POS	0.008	−2.505	1.663
3-hydroxy-cis-5-tetradecenoylcarnitine	NEG	0.003	−3.440	1.720

ESI, electrospray ionization; POS, positive ions; NEG, negative ions. The *P* were determined using student’s *t*-test. FC, fold changes (LRC VS HRC). Calculate the variable importance in the projection (VIP) value from Partial Least Squares-Based Discriminant Analysis (PLS-DA).

### 3.6 Metabolite KEGG enrichment analysis

**Figure 5:**
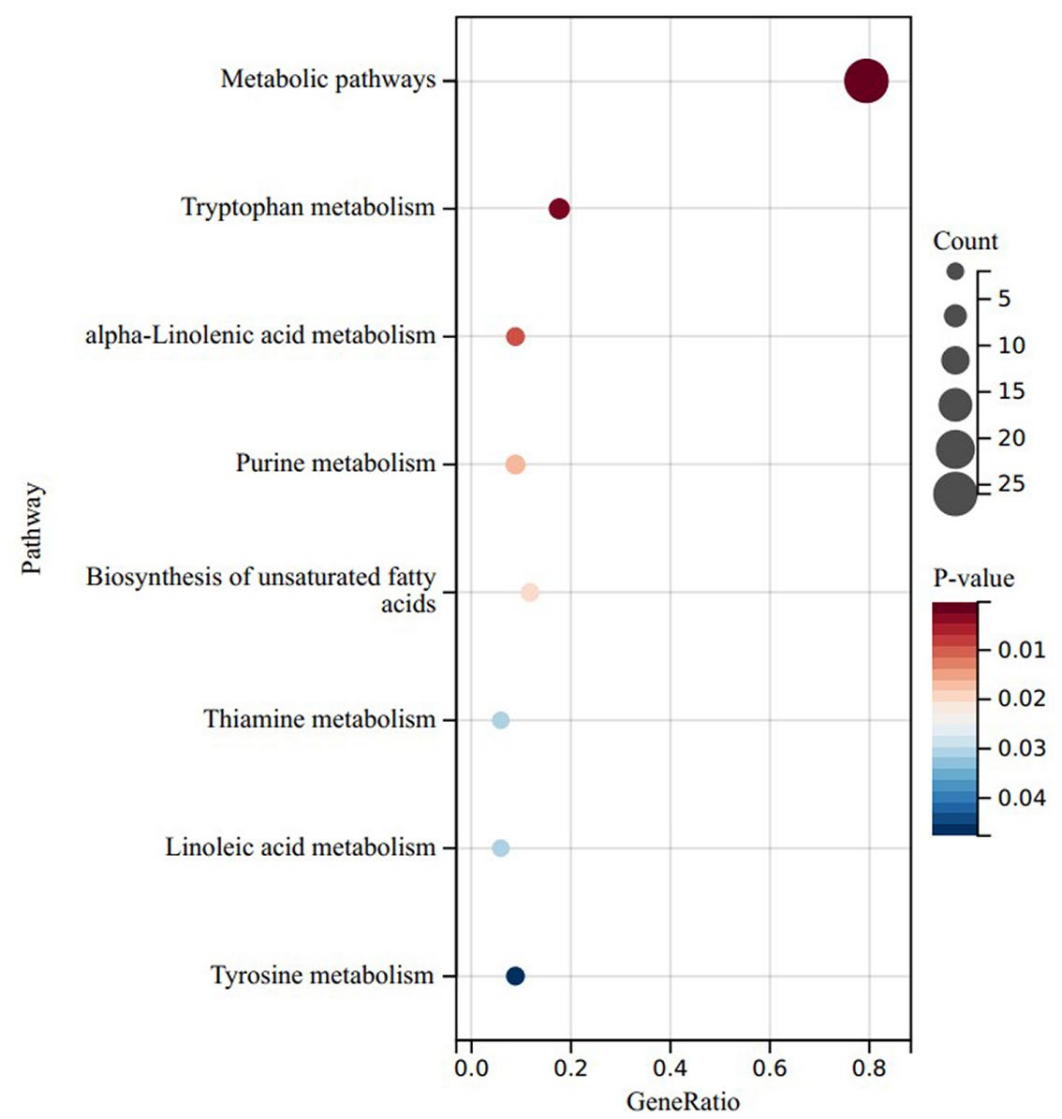
KEGG results of significantly upregulated differential enrichment in LRC group. *Y*-axis: enriched KEGG pathways based on differential metabolites; *X*-axis: target metabolites enriched relative to the total number of target metabolites in the respective pathway; Bubble area size: number of metabolites enriched; Bubble color: reflecting the significance of enrichment, where the color intensity represents the *P* magnitude. Redder hues indicate larger *P*, while bluer hues indicate smaller *P*.

To delve deeper into the functionalities of these divergent metabolites, we conducted a KEGG enrichment analysis on the metabolites that exhibited noteworthy upregulation and downregulation in the LRC group in comparison to the HRC group. Surprisingly, the upregulated differentials were primarily concentrated within metabolic pathways, tryptophan metabolism, alpha-linolenic acid metabolism, linoleic acid metabolism, thiamine metabolism, and biosynthesis of unsaturated fatty acids (*p* < 0.05) (Figure 5). The down-regulated differential metabolites were mainly enriched in metabolic pathways, but not significantly (*p* > 0.05).

### 3.7 Correlation between the metabolome and gut microbiome

**Figure 6:**
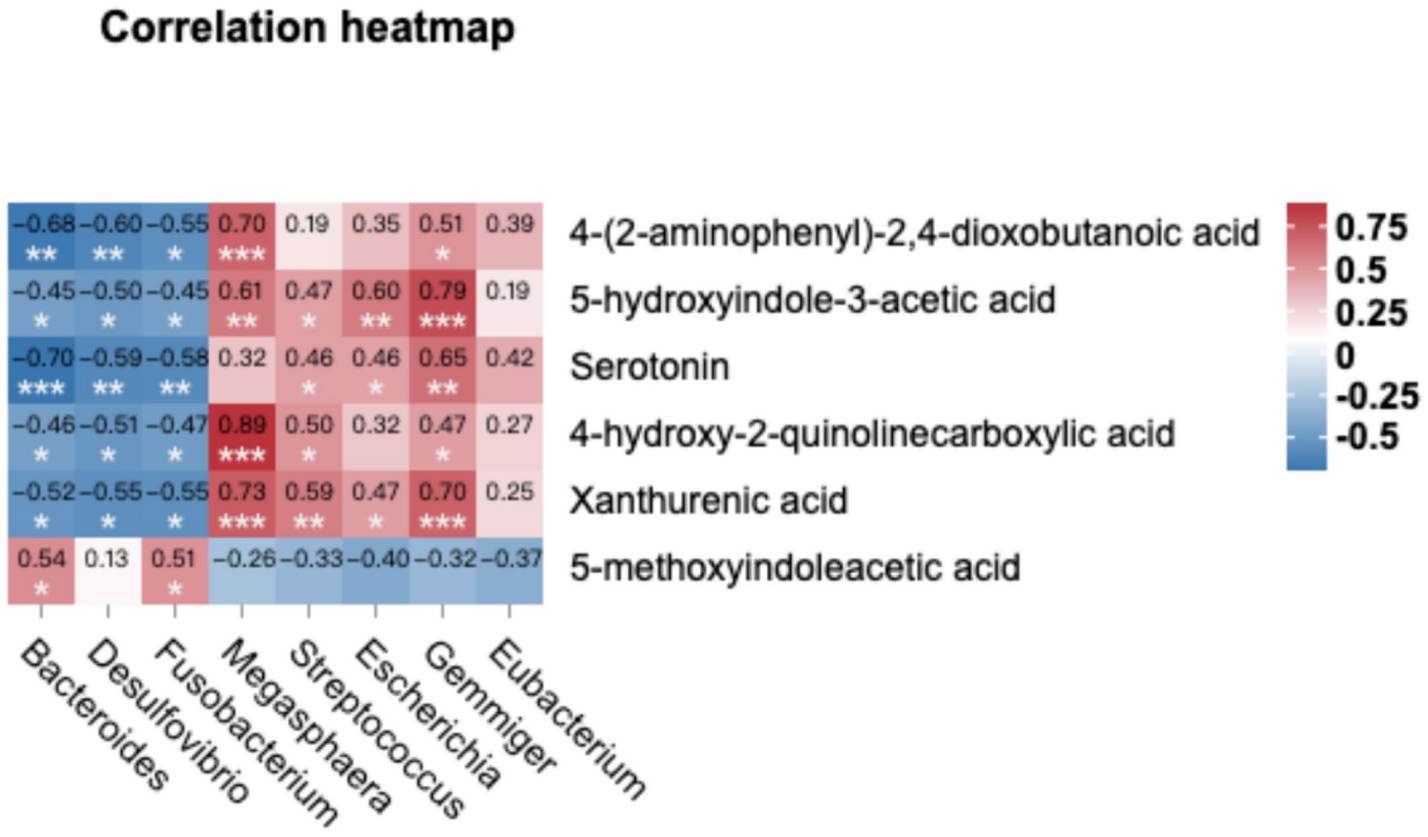
Combined metabolome and microbiome analysis. The Spearman correlation analysis was conducted to calculate the correlations between microbial genera (LDA > 4) and tryptophan metabolism-related metabolite. Positive correlations are depicted in red, while negative correlations are displayed in blue (***p < 0.001, **0.001 < *p* < 0.01, and *0.01 < *p* < 0.05).

We compared the KEGG enrichment results of the two omics and found that they were both enriched in tryptophan metabolism. Specifically, six differential metabolites were identified as enriched in the tryptophan metabolic pathway. These metabolites include 4-(2-aminophenyl)-2,4-dioxobutanoic acid, 5-hydroxyindole-3-acetic acid, xanthurenic acid, 4-hydroxy-2-quinolinecarboxylic acid, serotonin, and 5-methoxyindoleacetic acid (as listed in Table 6). In order to explore deeper into the relationship between metabolites and microorganisms, we conducted a Spearman correlation analysis between the metabolites enriched in the tryptophan metabolic pathway (identified through the KEGG analysis) and the differential microbial genera (identified with LDA score > 4 through LEfSe analysis). The correlation analysis revealed noteworthy associations, as illustrated in Figure 6. Specifically, 4-(2-aminophenyl)-2,4-dioxobutanoic acid and 5-hydroxyindole-3-acetic acid both correlated positively with *Megasphaera* and *Gemmiger* respectively, as did 4-hydroxy-2-quinolinecarboxylic acid and xanthurenic acid with *Megasphaera*. Conversely, serotonin showed a pronounced negative correlation with *Bacteroides*, underscoring an inverse relationship between them (*p* < 0.001).

**Table 6:** Tryptophan metabolism-related metabolite.

Name	ESI	Log_2_FC	VIP	*p*-value
4-(2-aminophenyl)-2,4-dioxobutanoic acid	NEG	7.254	1.323	0.030
5-hydroxyindole-3-acetic acid	NEG	4.431	1.345	0.027
Xanthurenic acid	POS	7.060	1.763	0.005
4-hydroxy-2-quinolinecarboxylic acid	POS	6.848	1.460	0.024
Serotonin	POS	1.666	2.425	0.000
5-methoxyindoleacetic acid	POS	−1.450	1.732	0.006

ESI, electrospray ionization; POS, positive ions; NEG, negative ions. The *P* were determined using student’s *t*-test. FC, fold changes (LRC VS HRC). Calculate the variable importance in the projection (VIP) value from Partial Least Squares-Based Discriminant Analysis (PLS-DA).

## 4 Discussion

The act of nourishment is one of the most fundamental and conservative activities in the animal kingdom. Regulating the intake of food is an essential process for animal survival (33). A multitude of prior investigations have found associations between feeding behavior and social order (34–36). Steffen Hoy’s research revealed that swine of higher grades were bestowed with lesser frequency of feedings at automated nourishment stations, exhibiting prolonged presence at the trough and displaying a heightened consumption of feeding (37). In this experiment, we refrained from directly quantifying the social standing of the subjects. Nonetheless, based on the observed feeding behavior, we postulate that swine with lower RFI potentially occupy a higher social status, characterized by extended occupation of automated feeding stations but with decreased frequency and decreased efficiency of feeding. On the contrary, lower social status entails a contrasting scenario, where pigs are frequently displaced from the feeding trough, requiring more frequent visits to obtain sustenance. Interference from other pigs disrupts their feeding process, forcing them to consume food hastily and consequently resulting in an increased average intake per minute. Our findings align with those of Hoy et al. (37). However, divergent outcomes have been reported in other studies. Herrera-Cáceres et al. (38) found that dominant animals fed more frequently. Nielsen et al. (39, 40) found no relationship between feeding behavior and social class. Hence, the relationship between feeding behavior and social class is not steadfast and can be influenced by various factors such as group size (40), kinship (41), strain (42), and among others.

Accumulating evidence suggests that the microbiota within the gastrointestinal system may influence the eating behavior of animals. In an examination of human feeding behaviors, it was discovered that *Clostridium XVIII* exhibited an association with more salubrious dietary practices and diminished subjective feelings of hunger (43). Simultaneously, *Fusobacterium* was found to be linked to unfavorable dietary attributes and elevated subjective sensations of hunger (43). This observation aligns with our own findings, indicating that swine with lower RFI exhibit unique feeding habits and consume less feed. Gastrointestinal bacteria might also play a role in the physiological regulation of the host’s appetite. For instance, *Escherichia* has the ability to generate caseinolytic protease B, a bacterial protein that resembles the α-melanocyte-stimulating hormone, thereby directly promoting satiety (44). Peptide YY (PYY), synthesized by enteroendocrine L cells, possesses a prominent function in the regulation of gastrointestinal processes and satiety. Studies conducted on mice lacking PYY gene expression revealed an enhanced abundance of Firmicutes along with a reduction in Bacteroidetes when compared to their wild-type counterparts (45). The fermentation process of dietary fiber by Firmicutes within the intestinal tract yields short-chain fatty acids (SCFA), including butyric acid, propionic acid, and acetic acid. These SCFAs can induce PYY secretion and exert influence on the brain, thereby evoking a sensation of satiety (46). In the cecal microbial composition of pigs with lower RFI, there is an elevated level of Firmicutes and a lower level of Bacteroidetes compared to pigs with higher RFI. This could potentially result in differences in subjective hunger perception among pigs, with low RFI pigs experiencing a greater sense of satiety.

Omega-3 (ω-3) and Omega-6 (ω-6) polyunsaturated fatty acids (PUFAs) are indispensable to animals, known for improving reproductive performance, immune regulation, enhancing intestinal functionality and improving meat quality (47–49). They hold a breadth of research potential in the food additive industry. In our study, we observed a significant increase of Alpha-Linolenic acid (ALA) (an ω-3 PUFA) and Linoleic acid (LA) (an ω-6 PUFA) in the LRC group. Both ALA and LA are indispensable fatty acids in swine nutrition, deriving exclusively from dietary sources. These fatty acids undergo conversion processes within the animal’s body, facilitated by the enzymatic actions of dehydrogenase and carboxylase, resulting in the synthesis of Arachidonic Acid, Eicosapentaenoic Acid, Docosahexaenoic Acid, and various other derivatives (50, 51). These highly active substances are capable of regulating physiological and biochemical responses in the organism, hence influencing both the endocrine and digestive systems. It has been found that the incorporation of ALA in the daily feed of lactating sows can boost the immunity and intestinal health of the piglets (47). Moreover, the addition of 10% Perilla cake (mainly composed of 72.7% unsaturated fatty acids, with ALA constituting between 55–64%) to the feed of growing pigs can significantly enhance daily weight gain (52). Another investigation discerned that the incorporation of 10–15% flaxseed oil, abundant in ALA, could enhance the feed conversion ratio and the back-fat composition of growing-finishing swine (53). Moreover, ALA can feasibly stimulate the expression of insulin like growth factor-1 (IGF-1) via the peroxisome proliferators-activated receptors (PPARs) signaling pathway, insinuating a crucial role it might play in facilitating swine growth and development (54). Incorporating 1% of LA into the daily ration may uphold the health of squabs by enhancing their antioxidative capabilities and lipid metabolic functions (55). As a finale to our discourse, it emerges that polyunsaturated fatty acids could be instrumental in augmenting the efficaciousness of swine feed efficiency, within which Alpha-Linolenic Acid and Linoleic Acid might fulfill pivotal roles.

Serotonin, a significant neurotransmitter, is synthesized from tryptophan via tryptophan hydroxylase, transforming into 5-hydroxytryptophan, which is subsequently converted within the enterochromaffin cells via 5-hydroxytryptophan decarboxylase. Although enterochromaffin cells are responsible for over 90% of serotonin production in the body, the gut microbiota is thought to influence the serotonergic system of the host gastrointestinal tract (56). For example, Reigstad et al. (57) demonstrated that intestinal flora promotes serotonin production through the effects of short-chain fatty acids on enterochromaffin cells. The impact of serotonin on feeding behavior has been the subject of extensive research (58), Serotonin has been found to inhibit feeding behavior by amplifying satiety signals and prolonging their duration (59). Because of the blood–brain barrier, peripheral serotonin theoretically cannot directly affect the brain. Peripherally located serotonin may respond to chemical and mechanical stimuli by releasing it into incoming nerve terminals, thereby initiating gastrointestinal reflexes and regulating visceral perception (60). Empirical evidence suggests that a peripheral injection of serotonin can expedite the onset of satiety in rats in a behaviorally specific manner, a mechanism that necessitates the concurrent operation of gastrointestinal mechanisms (61, 62). Considering the low feeding frequency observed in the LRC group, it is plausible to suggest that the high expression of serotonin might be associated with this phenomenon. *Escherichia*, *Streptococcus*, *Candida*, etc. are the main bacterial groups that make serotonin (63, 64), while *Bacteroides* has been reported to deplete serotonin (65). Reportedly, *Eubacterium* evinces the ability to produce butyrate (66). This butyrate, in turn, instigates the binding between Zinc-binding protein-89 (ZBP-89) and Tryptophan hydroxylase 1 (THP1) gene promoters, escalating the expression of the THP1 gene and subsequently elevating the levels of serotonin within the intestinal tract (57, 67). Therefore, variations in serotonin concentration among different groups may be attributable to differences in gut microbial composition. In the LRC group, *Escherichia* and *Eubacterium* in the gut may contribute to higher serotonin levels, whereas *Bacteroides* may exert a depleting effect on serotonin levels. Serotonin can improve pigs’ satiety, thereby reducing feed waste and improving feed efficiency.

In summary, we found that there are differences in the feeding behavior of pigs with different RFIs. Pigs with lower RFI were characterized by occupying the feeding station for longer time, but feeding frequency and efficiency were lower. *Clostridium_XVIII*, which was related to satiety, was enriched in pigs with high FE, while *Fusobacterium*, which was related to hunger, was more abundant in pigs with low FE. Polyunsaturated fatty acids may help improve pig feed efficiency, with α-linolenic acid and linoleic acid likely playing key roles. We discerned a conspicuous correlation between the tryptophan metabolic pathway and FE, where divergences in serotonin levels could be attributed to individual gut microbiota variations. Such insights could better facilitate our comprehension of the interrelation between gut microbiota and feed efficiency, though the reasons and mechanisms engendering these interactions warrant further corroboration.

## Data availability statement

The original contributions presented in the study are publicly available. This data can be found here: FigShare, https://doi.org/10.6084/m9.figshare.25708839.v1.

## Ethics statement

The animal study was approved by all experimental procedures were performed by the Institutional Review Board (IRB14044) and the Institutional Animal Care and Use Committee of the Sichuan Agricultural University under permit number DKY-B20140302. The study was conducted in accordance with the local legislation and institutional requirements.

## Author contributions

SW: Data curation, Formal analysis, Visualization, Writing – original draft, Writing – review & editing. DC: Conceptualization, Investigation, Software, Writing – review & editing. XJ: Conceptualization, Investigation, Software, Writing – review & editing. QS: Data curation, Methodology, Supervision, Writing – review & editing. YY: Formal analysis, Project administration, Validation, Writing – review & editing. PW: Writing – original draft, Writing – review & editing, Data curation, Supervision. GT: Funding acquisition, Resources, Visualization, Writing – review & editing.

## Footnotes

^1^ www.metaboanalyst.ca
